# Determinants of intention to use generative AI fitness assistants: Integrating the Theory of Planned Behavior, second-order information system quality, and perceived value

**DOI:** 10.1371/journal.pone.0353384

**Published:** 2026-07-09

**Authors:** Chongyang Li, Yaya Shi

**Affiliations:** 1 Graduate School, Jilin Sport University, Changchun, China; 2 Department of Physical Education, Yan’an University, Yan’an, China; East China Normal University, CHINA

## Abstract

As generative artificial intelligence (GAI) becomes embedded in sport and health management, GAI fitness assistants can generate and iteratively refine training plans through conversational interactions; however, their adoption remains shaped by users’ trade-offs between perceived quality and risk. Drawing on the Information Systems Success Model and the Theory of Planned Behavior (TPB), and incorporating perceived value, this study analyzed cross-sectional, self-reported data from 342 valid questionnaires using structural equation modeling to examine the relationships among second-order information system quality, subjective norms, attitude, perceived behavioral control, and category-level behavioral intention to use GAI fitness assistants. The results indicate that information system quality was significantly associated with more favorable attitude (β = 0.623) and perceived behavioral control (β = 0.485), with system quality contributing the greatest weight (0.805). Bootstrapped indirect-effect tests showed that subjective norm was significantly associated with behavioral intention through attitude, perceived behavioral control, perceived value, and the serial attitude-to-value pathway, whereas its direct effect was not significant. Perceived value is the strongest predictor of intention (β = 0.447), and attitude (β = 0.275) and perceived behavioral control (β = 0.163) are also important antecedents. Overall, the model explains 50.9% of the variance in intention to use. These findings suggest theoretical and practical implications for improving the stability of GAI fitness assistants, the reliability of their recommendations, and the communication of user value.

## 1. Introduction

Regular physical activity is essential for physical and mental health and for the prevention and control of chronic diseases. However, exercise management in the general population continues to face persistent challenges, including limited access to professional guidance, difficulty maintaining long-term engagement, and delayed or insufficient feedback, particularly among older adults and individuals with chronic conditions [1–4]. Although mobile health applications and wearable devices have shown promise in promoting physical activity through self-monitoring, feedback, and goal setting, most conventional fitness apps remain largely rule-based and dependent on templated content [1,5–7]. Consequently, when users’ goals change dynamically, constraints become complex, or ongoing adjustment is required during training, these apps often struggle to provide truly adaptive, individualized support or coach-like interaction [8].

With advances in natural language understanding and content generation, generative artificial intelligence (GAI) has begun to reshape digital fitness services from functional digitalization toward interactive content generation [9–13]. In this study, GAI fitness assistants are defined as digital fitness-support tools that use generative AI, usually through conversational or multimodal interfaces, to interpret users’ stated goals, physical constraints, and feedback; generate or revise exercise plans; explain training decisions; and provide motivational or educational support. Unlike traditional fitness apps, such assistants can generate and iteratively refine training plans, phased goals, and accompanying explanations on demand, thereby offering more context-sensitive guidance in scenarios involving fluctuating training time, changing goals, complex constraints, or the need for ongoing load adjustment [14,15]. Commercially announced or deployed examples include WHOOP Coach and Fitbit’s personal health coach built with Gemini. These examples are provided solely to contextualize the technology category; they were not named in the questionnaire, and respondents were not asked to evaluate any specific commercial system.

Nevertheless, GAI fitness assistants differ from general information services because training plans and load recommendations are safety-sensitive and may have consequential spillover effects. Inappropriate suggestions regarding intensity, frequency, exercise selection, or recovery scheduling may lead to sports injuries, elevated chronic disease risk, and erosion of trust [16]. Therefore, users’ intention to use such assistants is likely to depend not only on perceived usefulness, but also on broader judgments of system quality, controllability, risk, and value [17]. Although the present survey did not measure churn or actual discontinuance, potential withdrawal from future use may occur when generated advice is inconsistent, privacy expectations are violated, subscription or device costs increase, wearable data are poorly integrated, or users cannot determine when advice should be verified by a human coach or clinician.

Although emerging studies have discussed the potential of generative AI for producing health and fitness recommendations, the psychological mechanisms underlying intention to use GAI fitness assistants—where training-plan generation and iterative refinement are central—remain insufficiently theorized and empirically tested from information-systems and behavioral-science perspectives [8]. Existing digital health and mHealth adoption research has largely relied on the Technology Acceptance Model or its extensions, while research on AI tool adoption has often focused on general settings and has seldom captured the experiential complexity introduced by generativity, conversational interaction, and multimodal feedback [18]. Recent studies on AI chatbot and generative AI health-assistant adoption further show that information quality, system quality, social norms, privacy concerns, and health-context barriers jointly condition adoption decisions [19–21]. Moreover, in high-variability and high-risk fitness contexts, evaluating system performance through a single quality dimension may be insufficient to capture users’ holistic perceptions of the overall service experience [22]. Therefore, grounded in the Information Systems Success Model, this study develops a second-order information system quality construct integrating information quality, system quality, and service quality. It further incorporates attitude, subjective norm, and perceived behavioral control from the Theory of Planned Behavior, and adds perceived value to capture users’ benefit–cost trade-offs. Accordingly, this study addresses three research questions:

RQ1, to what extent does second-order information system quality explain users’ attitude and perceived behavioral control?

RQ2, how are subjective norms indirectly associated with behavioral intention through attitude, perceived behavioral control, and perceived value?

RQ3, in the context of GAI fitness assistants, what is the most critical predictor of users’ final behavioral intention to use?

## 2. Literature review and hypotheses development

As generative artificial intelligence (GAI) technologies become embedded in health and fitness contexts, existing research has increasingly examined users’ adoption intention and future-use intention, because the real-world benefits of such intelligent systems depend partly on whether users are willing to integrate them into daily health management routines [23]. As an important innovation in the digital health domain, GAI fitness assistants can understand users’ goals and constraints through natural language interaction and generate personalized training recommendations and plans, thereby potentially reshaping service models for traditional fitness guidance and personal health management [24]. Therefore, systematically identifying and explaining the key factors that shape users’ intention to use is essential for promoting the responsible diffusion of GAI fitness assistants in personalized training management [25].

### 2.1 Theoretical background

As digital health and intelligent technologies have continued to be embedded in sport and health management, researchers adopted and developed a range of established theoretical frameworks to explain users’ adoption, use, and future-use intentions toward related technologies [26,27]. For example, Chiu et al. [28] employed the expectation-confirmation model (ECM) to investigate factors influencing users’ intentions to continue using health and fitness applications. Similarly, Palos-Sanchez et al. [29] applied the technology acceptance model (TAM) to analyze individuals’ behavioral intention to use mobile health technologies. More recently, Li et al. [30] integrated the Theory of Planned Behavior (TPB) with the Health Belief Model (HBM) to test how attitude, subjective norms, perceived behavioral control, and health-belief-related factors affected exercise intention when users engaged with fitness mobile applications. Other frameworks, such as the Unified Theory of Acceptance and Use of Technology (UTAUT) and the task-technology fit (TTF) model, were also commonly used in health management technology research to characterize the roles of social influence, facilitating conditions, and the alignment between technological functionalities and task requirements in shaping intention [31,32].

GAI technologies are beginning to reshape the landscape of fitness services and to encourage traditional fitness models to undergo digital transformation [33]. Fitness enthusiasts are among the early groups encountering emerging GAI technologies capable of generating personalized training plans, but the present study does not assume that respondents used a particular commercial system. Therefore, examining users’ attitudes and intention to use this technology category when they perceived it as a high-quality information system was important. DeLone and McLean [34] noted that analyses of information system success had to consider quality dimensions and their effects on user satisfaction and intention. Given that GAI fitness assistants were inherently complex information systems integrating content generation, interactive services, and risk-laden outcomes, a single adoption perspective was insufficient to fully explain users’ decision-making processes. Accordingly, combining the holistic quality evaluation emphasized by the Information Systems Success Model (IS Success Model) with the psychological decision mechanisms proposed by the Theory of Planned Behavior (TPB), and further incorporating a perceived-value perspective capturing benefit-cost trade-offs, enabled a more systematic explanation of how users formed intentions to use GAI fitness assistants.

The Information Systems Success Model (IS Success Model), proposed by DeLone and McLean [34], provided a well-established framework for explaining how information system quality is associated with system success through user satisfaction and usage intention. The model highlighted the importance of information quality, system quality, and service quality (i.e., second-order information system quality) as key antecedents.

**Fig 1:**
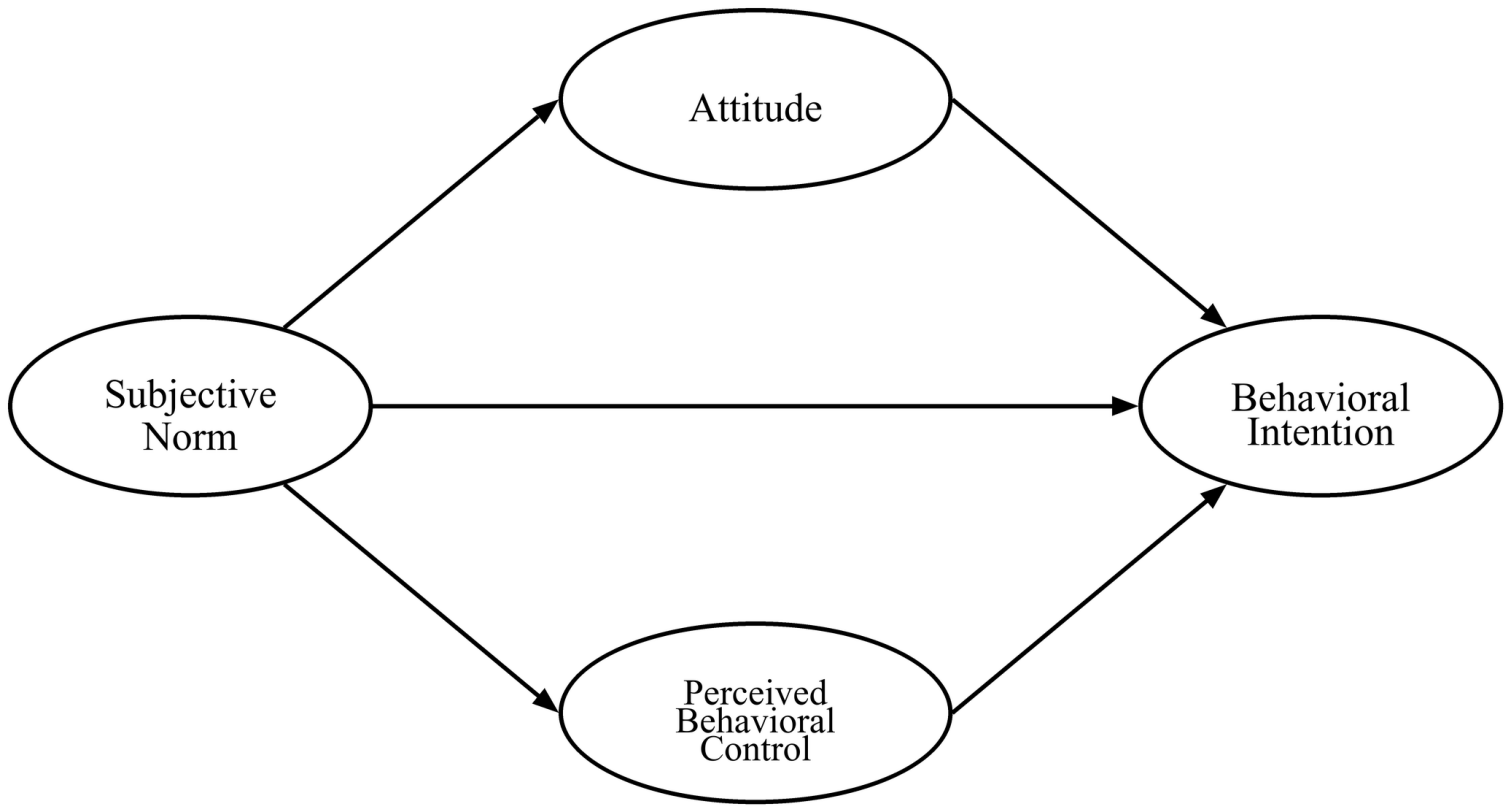
TPB theoretical model.

Meanwhile, the Theory of Planned Behavior (TPB) (see Fig 1) was extended from the Theory of Reasoned Action (TRA) to address the limitations of the original model in explaining behaviors that were not entirely under volitional control [35–37]. Ajzen [37] argued that behavioral intention was the most proximal antecedent of behavior, reflecting the extent of effort an individual was willing to invest in performing a behavior; whether the behavior occurred also depended on actual behavioral control, which was shaped by resources and opportunities such as time, money, skills, and cooperation from others. TPB further posited that behavioral intention was jointly predicted by attitude, subjective norm, and perceived behavioral control. In the context of GAI fitness assistants, this implied that users were more likely to report stronger intention to use when they held a more favorable evaluation of using the assistant, perceived support from important others, and believed they possessed the necessary resources and capabilities [38].

In addition, perceived value (PV) provided a critical complement for explaining technology adoption in high-risk contexts characterized by strong trade-offs. Compared with focusing only on usefulness or attitudinal tendencies, perceived value emphasized that individuals made an overall judgment about whether a technology was worth using by weighing expected benefits against expected costs [39,40]. In the context of GAI fitness assistants, benefits could be reflected in more personalized and efficient training recommendations, emotional support derived from continuous companionship, and cost savings by partially substituting for personal training services; costs included learning and interaction effort, concerns about privacy and data security, and physical risks arising from incorrect recommendations [41]. Therefore, even when users held a favorable attitude toward the technology, their intention to use could still decrease due to cost–risk trade-offs. Conversely, when users perceived that overall benefits substantially outweighed the costs and risks, perceived value became a direct predictor of intention to use. Within the integrated framework of this study, information system quality and the three TPB components jointly shaped users’ evaluations of benefits and costs and further influenced behavioral intention through perceived value.

Although TPB and the IS Success Model had been widely applied in studies of mobile health and general apps, research that simultaneously employed second-order information system quality and TPB mechanisms to explain intention to use generative AI fitness assistants remained relatively limited. More importantly, in the high-variability and high-risk context of fitness recommendation generation, users often did not make decisions solely based on attitude or social influence; instead, they further weighed benefits against costs to form an overall judgment of whether the technology was worth using [42,43]. Therefore, it was necessary to reveal how second-order information system quality (a technological attribute) worked through attitude, subjective norm, and perceived behavioral control (psychological attributes), and further via perceived value as a trade-off-based evaluative mechanism, to jointly influence intention to use. Accordingly, this study adopted an integrative three-theory perspective to develop and test the formation mechanism underlying users’ intention to use GAI fitness assistants, thereby addressing this gap.

In summary, this study used the IS Success Model to characterize the holistic quality foundation of GAI fitness assistants, applied TPB to explain the psychological pathways through which intention was formed, and incorporated perceived value to capture the decision outcome of benefit-cost trade-offs, thereby constructing an integrated framework that more closely reflected category-level intention formation in this emerging technology context.

### 2.2 Hypotheses development

**Fig 2:**
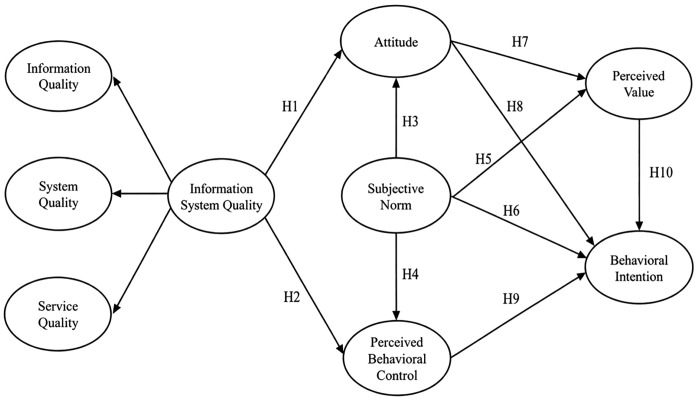
Theoretical model.

Prior research had demonstrated that the Theory of Planned Behavior (TPB) was an important framework for explaining health technology adoption and designing behavior-change interventions. However, in the context of GAI fitness assistants—which emphasized conversational interaction, training safety, and sustained engagement—relying solely on attitude, subjective norm, and perceived behavioral control was insufficient to capture users’ judgments of overall system performance and their benefit–cost trade-offs. Therefore, building on TPB, this study incorporated second-order information system quality from the Information Systems Success Model and introduced the construct of perceived value, thereby forming an extended integrative model and proposing corresponding hypotheses (see Fig 2).

#### 2.2.1 Information system quality.

In information systems research, quality dimensions had been regarded as key antecedents of users’ evaluations and behavioral responses. In their updated Information Systems Success Model, DeLone and McLean [34] emphasized that system quality, information quality, and service quality constituted the core basis for users’ judgments of a system, and that these quality perceptions further shaped overall evaluations and subsequent usage tendencies by influencing use-related responses and satisfaction. In a comprehensive review of empirical studies published between 1992 and 2007, Petter et al. [44] noted that these quality dimensions represented one of the most frequently used and most consistently supported sets of constructs for explaining information system success outcomes. Accordingly, this study conceptualized information system quality as a second-order construct that subsumed three first-order dimensions—information quality, system quality, and service quality—to capture users’ holistic perceptions of the overall quality of GAI fitness assistants.

First, information quality and system quality were directly related to users’ perceptions of the credibility of generated content and the reliability of the interaction experience. In a health information context, Yi et al. [45] found that perceived information quality served as an important source of initial trust formation, and that trust further promoted more favorable overall evaluations. In health-related online services, Shim and Jo [46] similarly showed that information quality, system quality, and service quality jointly enhanced user satisfaction and perceived benefits, thereby improving users’ overall judgments. When GAI fitness assistants consistently generated more accurate and relevant training recommendations and maintained stable and timely responses, users were more likely to develop a positive attitude.

Second, system quality and service quality could also strengthen users’ sense of control by reducing operational burden and providing supportive resources. Ajzen [47] argued that perceived behavioral control was closely associated with individuals’ judgments about the feasibility of performing a behavior, encompassing both controllability and capability beliefs akin to self-efficacy. Compeau and Higgins [48] further indicated that self-efficacy constituted an important psychological foundation for technology use and was influenced by support conditions and prior usage experience. As conversational health technologies became increasingly prevalent, Laranjo et al. [49] emphasized that usability and interaction capabilities were central considerations in the adoption and evaluation of such systems in health contexts. Therefore, when a GAI fitness assistant provided clearer guidance, greater ease of use, and more timely support services, users were more likely to believe they had sufficient resources and capabilities to use it effectively, thereby increasing perceived behavioral control. Based on the above analysis, the following hypotheses were proposed:

H1: Information system quality positively affected attitude.

H2: Information system quality positively affected perceived behavioral control.

#### 2.2.2 Subjective norm.

Subjective norm referred to the support and pressure an individual perceived from important others or relevant social groups when deciding whether to perform a behavior. Ajzen [37] argued that normative beliefs aggregated into subjective norm, which, together with attitude and perceived behavioral control, determined behavioral intention. In the context of GAI fitness assistants, suggestions from family and friends, discussions with workout partners, and prevailing views in online communities could all serve as important social cues shaping users’ decisions.

Regarding attitude formation, approval from social groups could change individuals’ evaluations through normative and informational influences. Venkatesh and Davis [50] indicated that social influence processes could enhance individuals’ perceptions of a technology’s usefulness and further facilitate the formation of adoption tendencies. When users were continuously exposed to positive experiences and recommendations about GAI tools within fitness communities, their risk-related associations were more likely to be attenuated, thereby fostering a more favorable attitude toward using the tool.

In terms of perceived behavioral control, subjective norm often manifested not only as attitudinal support but also as access to external resources. In organizational settings, Venkatesh and Morris [51] found that subjective norm played a role in technology use decisions and that this effect changed over time and with experience, suggesting that peer influence often co-occurred with learning support and adaptation processes. Therefore, when peers provided operational experience, usage tips, and problem-solving suggestions, users were more likely to perceive that they possessed sufficient ability and conditions to use GAI fitness assistants, thereby increasing perceived behavioral control.

With respect to perceived value, subjective norm can increase users’ value judgments through social endorsement, informational influence, peer learning, and reduced trial-and-error costs. In an emerging category such as GAI fitness assistants, many users lack clear benchmarks for judging whether generated plans are safe, personalized, and worth the effort of learning new interaction routines. Support from important others, demonstrations by peers, and guidance from teachers or coaches can signal that the assistant’s expected benefits outweigh learning effort, subscription or device costs, privacy concerns, and safety risks. These social cues may also provide reusable prompts, examples of iterative plan refinement, and risk reminders, thereby lowering the cost of exploration and improving the perceived return on use. Prior technology-adoption research has shown that social influence shapes perceived usefulness and intention [50,51], and recent generative AI adoption studies in education similarly report that social norms operate alongside attitude, perceived control, trust, and AI literacy [38,52]. Although the direct effect of subjective norm is sometimes weak or non-significant in technology contexts, it was retained here because GAI fitness assistants involve visible peer discussion, teacher or coach endorsement, and perceived social expectations around responsible digital-health practices. Based on the above analysis, the following hypotheses were proposed:

H3: Subjective norm positively affected attitude.

H4: Subjective norm positively affected perceived behavioral control.

H5: Subjective norm positively affected perceived value.

H6: Subjective norm positively affected intention to use.

#### 2.2.3 Attitude.

Attitude referred to an individual’s overall positive or negative evaluation of performing a specific behavior and had been regarded as one of the most stable psychological predictors of behavioral intention. Within the TPB framework, Ajzen [37] argued that attitude stemmed from individuals’ beliefs about behavioral outcomes and their value appraisals, and it functioned as a direct antecedent of intention formation. In the context of GAI fitness assistants, attitude was reflected in users’ overall assessment of whether the training recommendations were useful, whether the interaction was enjoyable, and whether the use process elicited positive emotional experiences.

Although attitude and perceived value are related, they are conceptually distinct in this study. Attitude refers to users’ favorable or unfavorable evaluation of using GAI fitness assistants, including cognitive judgments of usefulness and affective reactions such as enjoyment or interest. Perceived value, by contrast, refers to a benefit-sacrifice calculus: whether the expected gains from personalized training guidance, time savings, and motivational support outweigh sacrifices such as learning effort, privacy concerns, subscription or device costs, and potential exercise-safety risks [39,40]. Attitude is therefore modeled as an antecedent of perceived value because favorable evaluations can increase the salience of expected benefits and reduce the perceived burden of sacrifices, while perceived value remains a separate utility judgment.

Moreover, the motivating role of attitude in shaping intention to use has received empirical support in recent studies on generative AI. For example, research on generative AI adoption in higher education has shown that attitude is a central predictor of behavioral intention and interacts with factors such as AI literacy, self-efficacy, and trust [38,52,53]. Research on generative AI chatbot services also indicates that perceived competence and warmth can enhance service value and positive user responses [54]. In the GAI fitness context, a positive attitude toward conversational guidance, iterative plan refinement, and understandable explanations should therefore increase perceived value while also directly strengthening intention to use. Based on the above analysis, the following hypotheses were proposed:

H7: Attitude positively affected perceived value.

H8: Attitude positively affected intention to use.

#### 2.2.4 Perceived behavioral control.

Perceived behavioral control (PBC) reflected users’ subjective judgments about the capabilities and resources required to perform a specific behavior, with a core emphasis on perceived controllability and self-efficacy. Ajzen [37] argued that PBC not only influenced behavioral intention but could also exert a direct effect on behavior when resource constraints were substantial.

In the context of GAI fitness assistants, users often needed a certain level of human–computer interaction competence (e.g., effectively articulating training goals, constraints, and feedback information), while also relying on external resources such as devices, network access, and sustained self-tracking. In their research on generative AI use, Lin and Wang [55] found that generative AI self-efficacy significantly increased intention to use, indicating that perceived capability served as an important psychological foundation for intention. Similarly, Sui et al. [56], when testing AIGC usage intention based on TPB, found that PBC had a direct positive effect on intention to use. In addition, Chen et al. [57] emphasized that external support could promote digital behaviors by enhancing PBC. This suggested that providing tutorials, example dialogues, and error-correction mechanisms on fitness platforms could help compensate for deficits in resources and skills, strengthen users’ sense of control, and ultimately increase intention to use. Based on the above analysis, the following hypothesis was proposed:

H9: Perceived behavioral control positively affected intention to use.

#### 2.2.5 Perceived value.

Perceived value represented users’ overall utility judgment formed after weighing benefits against sacrifices. Zeithaml [40] indicated that consumers evaluated the value of a product or service by comparing what they received with what they gave up.

For GAI fitness assistants, benefits were typically reflected in the accuracy and personalization of recommendations, time and money savings enabled by on-demand availability, and emotional support derived from interaction; sacrifices included learning and cognitive effort, privacy concerns, and the risk of incorrect recommendations. In a study of generative AI chatbot services, Casaló et al. [54] found that perceptions of competence and warmth significantly enhanced users’ functional and emotional value assessments. Hao et al. [58] also demonstrated that perceived value increased significantly when perceived benefits rose while complexity and risk declined, and that perceived value strongly predicted adoption intention. Therefore, when users perceived higher overall value in a GAI fitness assistant, they were more likely to report stronger intention to use. Based on the above analysis, the following hypothesis was proposed:

H10: Perceived value positively affected intention to use.

## 3. Methodology

### 3.1 Ethics statement

Ethical approval for this study was obtained from the School of Physical Education, Yan’an University. Before completing the questionnaire, all participants were informed of the purpose of the study, the anonymous nature of the survey, and their right to withdraw at any time without penalty. Electronic informed consent was obtained from all participants prior to participation. The study was conducted in accordance with the ethical principles of the Declaration of Helsinki.

### 3.2 Survey design

Given the lack of a generic scale specifically designed for GAI fitness assistants, this study adopted validated measurement items from related domains and contextualized them to ensure both comparability and theoretical traceability.

To reduce differences in respondents’ understanding of generative AI fitness assistants, this study presented a standardized visual scenario description at the beginning of the questionnaire, introducing the concept, core functions, and typical usage process of the target technology. The material described a generative AI fitness assistant as an intelligent digital tool that can understand users’ fitness goals, physical conditions, available time, and training feedback through text, voice, or multimodal interaction, and can generate, explain, and adjust personalized training plans. Respondents were instructed to answer the subsequent items based on this standardized hypothetical scenario rather than evaluating any specific commercial product, thereby improving the contextual consistency and interpretability of the questionnaire measurements.

Grounded in the Information Systems Success Model, the Theory of Planned Behavior (TPB), and perceived value, this study developed the questionnaire by combining the adoption of established scales with contextual adaptation. The overall procedure included scale selection, contextual rewriting, pilot testing, and item refinement, ultimately producing a formal measurement instrument for structural equation modeling (SEM).

Regarding measurement sources, the three first-order dimensions (information quality, system quality, and service quality) were measured using items primarily adapted from well-established and empirically validated scales widely used in the e-service quality and information systems literature, particularly those developed by Parasuraman et al. [59] and Nelson et al. [60]. The TPB constructs (attitude, subjective norm, and perceived behavioral control) were mainly adapted from the classic measures proposed by Ajzen [61], Fishbein and Ajzen [62], Van Lange et al. [63], and Compeau and Higgins [48]. Perceived value was primarily measured using items drawn from Kim et al. [39]. Behavioral intention was adapted from the scale developed by Taylor and Todd [64]. Based on these instruments, this study further added context descriptions related to generative AI (GAI) fitness assistants to enhance the coverage of the target technology and usage scenarios, ensuring that the meaning of each construct was adequately represented in the new technological context.

To enhance contextual consistency and participants’ comprehension, this study contextualized the wording of selected items by drawing on typical interaction patterns between users and generative AI fitness assistants in fitness coaching scenarios. Specifically, within the information system quality section, the items highlighted features related to generative interaction, including the stability and response speed of the conversational function, ease of use, and the extent to which generated recommendations were logical, clear, and actionable, thereby enabling participants to make judgments based on their category-level perceptions of such assistants. In the subjective norm section, the items focused on the influence of key referent groups relevant to participants’ daily study and exercise routines (e.g., parents, significant others, peers, and physical education teachers) on their usage decisions, thereby improving the fit of normative sources. The contextual adaptation process preserved the original theoretical meaning of each construct while replacing generic service-use wording with fitness-assistant scenarios. All revised items were reviewed during the pilot test for clarity, semantic overlap, and factor-loading performance. In the perceived value and behavioral intention sections, the items adopted a benefit-cost trade-off framing, emphasizing the relative gains of using the assistant in terms of time, effort, and input costs, and further assessing individuals’ future intention to use the technology category.

Regarding scale format, Brown [65] and Dawes [66] suggested that, compared with scales using fewer response categories, seven-point Likert scales typically demonstrated superior reliability and validity [65,66]. Therefore, this study used a seven-point Likert scale to measure all observed variables, with anchors ranging from 1 (strongly disagree) to 7 (strongly agree). The initial questionnaire contained 37 measurement items covering eight first-order latent variables, among which information quality, system quality, and service quality jointly formed the second-order latent construct of information system quality.

Before the formal survey administration, the research team conducted a pilot test and obtained 55 valid responses to assess item comprehensibility and preliminary measurement quality. Based on the results of reliability and validity analyses, two items (IQ3 and SER4) were removed due to low factor loadings and partial semantic redundancy. The remaining items were slightly refined in wording according to participant feedback. The final questionnaire comprised 35 measurement items (see S1 Appendix) and additionally included three demographic variables (gender, education level, and exercise frequency) for sample description and control analyses.

### 3.3 Data collection

The questionnaire was administered mainly from early January 2026 to late January 2026 through a professional online survey platform (Wenjuanxing, https://www.wjx.cn/). To ensure data integrity during distribution, the platform’s response-time tracking and IP restriction functions were enabled to prevent duplicate submissions. In total, 433 responses were collected from individuals with established fitness habits or an interest in digital health. Participants responded to a standardized category-level scenario and were not required to be verified current users of a named GAI fitness application.

Strict procedures were implemented to ensure sample quality, consistent with the methodological recommendations summarized by Hair et al. [67] and Wang et al. [68]. The screening process involved three steps. First, based on insights from the pilot test, completing the questionnaire typically required 2–6 minutes. Therefore, participants who finished the survey in an unusually short time (i.e., less than 120 seconds) were considered to have approached the task carelessly, and their responses were classified as invalid. Second, the questionnaire included an attention-check item (e.g., “Please select ‘5’ for this question”). Responses from participants who failed to answer this item correctly were treated as invalid to eliminate careless responding. Finally, response patterns were examined, and questionnaires in which the same option was selected for all items (e.g., choosing “7” for every question) were removed.

After screening, 91 invalid questionnaires were excluded. Specifically, 36 responses were discarded due to insufficient completion time, 36 were removed because of incorrect answers to the attention-check item, and 19 exhibited obvious straight-lining patterns. Consequently, 342 valid questionnaires were retained for formal data analysis, yielding an effective response rate of 79.0%. Given the 35 observed measurement items in the final questionnaire, the sample-to-item ratio was approximately 9.77:1, which is close to the commonly cited 10:1 guideline. Accordingly, the findings should be generalized cautiously beyond exercise-engaged and digitally interested potential users.

### 3.4 Data analysis

This study used SPSS 27.0 and AMOS 28.0 to conduct statistical analyses of the survey data. First, SPSS was used for data cleaning and descriptive statistics, as well as for preliminary reliability testing of the measurement items and profiling of sample characteristics. Subsequently, the proposed theoretical model was validated and the hypotheses were tested using covariance-based structural equation modeling (CB-SEM) in AMOS. The higher-order information system quality construct was specified reflectively because information quality, system quality, and service quality were treated as correlated manifestations of an overall perceived quality evaluation rather than as formative components that independently constitute the construct. Compared with variance-based approaches such as PLS-SEM, CB-SEM was chosen because it enabled the simultaneous estimation of relationships among latent variables and provided overall model-fit information, thereby better supporting statistical tests of the hypothesized model [67].

In terms of analytical procedure, a two-stage approach was followed [69]. In the first stage, the measurement model was evaluated by systematically assessing the reliability and validity of each latent construct to ensure stable and sound measurement quality. In the second stage, after the measurement model met the required criteria, the structural model fit was assessed and standardized path coefficients were estimated to test the hypothesized relationships. Once the reliability and validity tests are satisfied, model fit is then examined to ensure that the estimated paths accurately reflect the underlying relationships. Subsequently, the interrelationships among the different constructs are analyzed in depth [67].

## 4. Results

### 4.1 Common method bias

All questionnaire data in this study were obtained from the same source via respondents’ self-reports and were measured using a fixed response format. Therefore, the data might have been subject to common method bias (CMB). CMB was typically induced by factors such as a shared measurement context, item framing, and item characteristics, which could result in spurious covariance between predictor and outcome variables [52,70]. To mitigate this risk and reduce systematic error attributable to common methods, multiple procedural remedies were implemented during questionnaire design and administration. First, measurement items for different constructs were placed on separate pages to reduce consistency motives and same-source priming effects caused by item adjacency, while also providing brief buffering between pages to limit the accumulation of common method variance [71,72].

For statistical assessment, Harman’s single-factor test was conducted to evaluate potential CMB. The results showed that the first unrotated factor accounted for 33.786% of the total variance, which was below the commonly used 50% threshold [73,74]. This finding suggested that no single factor dominated the variance structure and that common method bias was unlikely to materially affect the findings.

### 4.2 Validity and reliability testing

Before conducting the structural equation modeling (SEM) analyses, this study first performed a systematic assessment of the reliability and validity of the measurement scales (see Table 1). For reliability, Cronbach’s alpha coefficients were calculated using SPSS and were cross-validated with composite reliability (CR). The results showed that Cronbach’s alpha values for all latent constructs ranged from 0.794 to 0.921, exceeding the recommended threshold of 0.70 [73], which indicated good internal consistency. Meanwhile, CR values ranged from 0.795 to 0.922, all above the 0.70 benchmark [75], further supporting the stability and reliability of the measures.

**Table 1:** The results of the reliability and convergent validity of the measurement model.

Dimension	Items	P	Std.	CR	AVE	Cronbach’s alpha
System Quality	SQ1		0.788	0.900	0.599	0.899
	SQ2	***	0.787			
	SQ3	***	0.730			
	SQ4	***	0.734			
	SQ5	***	0.806			
	SQ6	***	0.796			
Service Quality	SER1		0.786	0.879	0.594	0.878
	SER2	***	0.797			
	SER3	***	0.840			
	SER4	***	0.661			
	SER5	***	0.758			
Information Quality	IQ1		0.848	0.922	0.663	0.921
	IQ2	***	0.818			
	IQ3	***	0.806			
	IQ4	***	0.776			
	IQ5	***	0.830			
	IQ6	***	0.806			
Attitude	ATT1		0.824	0.884	0.656	0.883
	ATT2	***	0.838			
	ATT3	***	0.822			
	ATT4	***	0.753			
Perceived Value	VAL1		0.796	0.865	0.617	0.865
	VAL2	***	0.784			
	VAL3	***	0.758			
	VAL4	***	0.802			
Perceived Behavioral Control	PBC1		0.765	0.795	0.565	0.794
	PBC2	***	0.696			
	PBC3	***	0.791			
Subjective Norm	SN1		0.767	0.858	0.603	0.858
	SN2	***	0.794			
	SN3	***	0.781			
	SN4	***	0.763			
Behavioral Intention	BI1		0.876	0.896	0.743	0.897
	BI2	***	0.873			
	BI3	***	0.836			

Note: *** p < 0.001.

Because relying solely on Cronbach’s alpha was insufficient to fully demonstrate measurement quality, this study further examined convergent and discriminant validity. Convergent validity was primarily assessed using average variance extracted (AVE). The AVE values for all constructs ranged from 0.565 to 0.743, exceeding the 0.50 threshold [76], indicating that the latent variables explained a substantial proportion of variance in their indicators and that the measurement model demonstrated adequate convergent validity.

Regarding discriminant validity, the Fornell–Larcker criterion was applied by comparing the square roots of AVE with the inter-construct correlation coefficients [76]. As shown in Table 2, the bolded diagonal values represented the square roots of AVE for each construct (ranging from 0.752 to 0.862), whereas the off-diagonal values represented Pearson correlation coefficients among constructs. The results indicated that the square root of AVE for each construct was greater than its correlations with all other constructs, suggesting that each construct explained more variance in its own indicators than it shared with other constructs, thereby supporting adequate distinctiveness among the constructs. The largest inter-construct correlation was 0.697, which was below the commonly used threshold for severe construct overlap, further supporting discriminant validity. Although several moderate correlations were observed between constructs, the square roots of AVE for all constructs still exceeded the corresponding inter-construct correlations, indicating that discriminant validity remained at an acceptable level. Overall, the measurement model met commonly used criteria for discriminant validity in SEM research, providing a reliable measurement foundation for subsequent structural model analyses.

**Table 2:** Discriminant validity.

	AVE	BI	SN	PBC	VAL	ATT	IQ	SER	SQ
BI	0.743	**0.862**							
SN	0.603	0.344	**0.777**						
PBC	0.565	0.476	0.456	**0.752**					
VAL	0.617	0.664	0.479	0.428	**0.785**				
ATT	0.656	0.642	0.503	0.565	0.697	**0.810**			
IQ	0.663	0.147	0.412	0.414	0.322	0.489	**0.814**		
SER	0.594	0.346	0.127	0.36	0.316	0.481	0.467	**0.771**	
SQ	0.599	0.341	0.292	0.454	0.433	0.582	0.600	0.500	**0.774**

***Note:*** *The bold numbers on the diagonal represent the root of AVE, and the lower triangle represents the Pearson correlation coefficient of the facet.*

### 4.3 SEM fit indices

To avoid spurious associations among hypothesized paths resulting from model misspecification or sampling error, the overall SEM model fit was evaluated before testing the structural relationships (see Table 3). Following Hair et al. [67], threshold values suggested by Hayduk [77], Bagozzi and Yi [78], Hair et al. [79], and Hu and Bentler [80] were adopted as the evaluation criteria. Model fit indices were computed using Amos 28.0. The results showed that CMIN/DF = 1.292, GFI = 0.893, CFI = 0.978, TLI = 0.976, RMSEA = 0.029, and SRMR = 0.0495, all of which met or exceeded the recommended standards. Given that the chi-square statistic and degrees of freedom were sensitive to sample size, the combined evidence from multiple indices indicated that the model fit was satisfactory. These results suggested that the proposed model adequately fit the data and provided a robust basis for subsequent analyses of variable relationships and hypothesis testing.

**Table 3:** Standard model fit indices.

Fit indices	Model indicator values	Standard	Conclusion	Source
CMIN	705.231	Reported; no fixed cutoff	Affected by sample size; no specific cut-off is recommended	
DF	546	Reported; no fixed cutoff		
CMIN/DF	1.292	<5 Acceptable; < 3 Good fit	Good fit	Hayduk, 1987
GFI	0.893	>0.8 Acceptable; > 0.9 Good fit	Acceptable	Bagozzi & Yi, 1988
CFI	0.978	>0.8 Acceptable; > 0.9 Good fit	Good fit	Bagozzi & Yi, 1988
TLI (NNFI)	0.976	>0.8 Acceptable; > 0.9 Good fit	Good fit	Hair et al., 2017
RMSEA	0.029	<0.08	Good fit	Hair et al., 2017
SRMR	0.0495	<0.08	Good fit	Hu & Bentler, 1998

### 4.4 SEM validation

**Fig 3:**
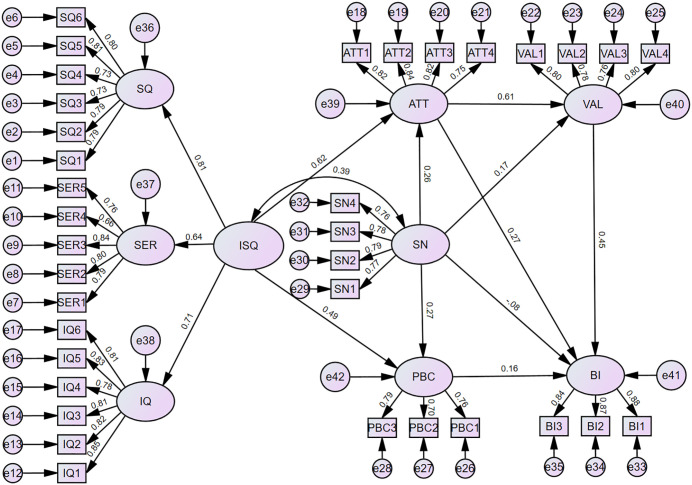
Structural model diagram.

This study estimated the structural model using AMOS (v28.0) to obtain standardized path coefficients and to further assess the explanatory power of the endogenous variables (see Fig 3). Given that information system quality (Information System Quality, ISQ) was modeled as a second-order construct, it was necessary to further examine the contribution weights of its first-order dimensions to the higher-order construct (see Table 4). The results showed that the loadings of system quality (SQ), service quality (SER), and information quality (IQ) on ISQ all reached statistical significance (all paths p < 0.001), indicating that these three dimensions jointly constituted a valid representation of overall information system quality.

**Table 4:** Weight distribution in second-order models.

Second-order variable	First-order variable	P	Loading
Information System Quality	Information System Quality → SQ	***	0.805
Information System Quality	Information System Quality → SER	***	0.639
Information System Quality	Information System Quality → IQ	***	0.710

In terms of contribution weights, the path ISQ → SQ exhibited the highest weight (0.805), followed by ISQ → IQ (0.710), whereas ISQ → SER was relatively lower (0.639). This pattern suggested that, in the formation of users’ perceptions of the overall quality of GAI fitness assistants, system-level performance and stability served as the most central source of quality, followed by the quality of informational content, while service support factors played a significant yet comparatively secondary role. Overall, the distribution of weights in the second-order model supported the specification of ISQ as a higher-order quality construct and provided a reliable measurement basis for subsequent structural path analyses.

To present the mechanisms among variables more clearly, this study further tested the hypothesized paths and reported the standardized path coefficients (β) and significance levels of the structural model (see Table 5). Regarding the formation of attitude (ATT), both subjective norm (SN) and information system quality (ISQ) exerted significant positive effects on attitude (SN → ATT, β = 0.260, p < 0.001; ISQ → ATT, β = 0.623, p < 0.001), with the effect of ISQ being stronger than that of SN. Taking ISQ → ATT as an example, the result could be interpreted as follows: when ISQ increased by one standard deviation, users’ attitude increased by approximately 0.623 standard deviations, indicating that overall system quality served as a key driver of positive attitude formation.

**Table 5:** Structural model path analysis results.

Hypothesis	Relationship			Ustd.	S.E.	C.R.	P	Std. (β)	R^2^
H3	SN	→	ATT	0.277	0.061	4.55	***	0.260	0.584
H1	ISQ	→	ATT	0.821	0.099	8.276	***	0.623	
H7	ATT	→	VAL	0.586	0.064	9.163	***	0.608	0.507
H5	SN	→	VAL	0.179	0.061	2.906	0.004	0.174	
H4	SN	→	PBC	0.268	0.066	4.081	***	0.270	0.412
H2	ISQ	→	PBC	0.595	0.095	6.267	***	0.485	
H8	ATT	→	BI	0.318	0.092	3.462	***	0.275	0.509
H6	SN	→	BI	−0.099	0.076	−1.304	0.192	−0.08	
H9	PBC	→	BI	0.204	0.078	2.627	0.009	0.163	
H10	VAL	→	BI	0.537	0.093	5.769	***	0.447	

Note. *** p < 0.001.

Regarding perceived value (VAL), both attitude and subjective norm exerted significant positive effects (ATT → VAL, β = 0.608, p < 0.001; SN → VAL, β = 0.174, p = 0.004). The effect of attitude was more pronounced, indicating that individuals’ favorable evaluations of GAI fitness assistants served as an important source of value judgments. For perceived behavioral control (PBC), subjective norm and information system quality also had significant positive effects on PBC (SN → PBC, β = 0.270, p < 0.001; ISQ → PBC, β = 0.485, p < 0.001), suggesting that subjective norm and overall system quality jointly enhanced users’ perceptions of whether they possessed sufficient capability and necessary conditions to use the assistant, thereby strengthening perceived behavioral control.

With respect to behavioral intention (BI), attitude, perceived behavioral control, and perceived value were all significant predictors (ATT → BI, β = 0.275, p < 0.001; PBC → BI, β = 0.163, p = 0.009; VAL → BI, β = 0.447, p < 0.001), with perceived value showing the largest effect. In contrast, the direct effect of subjective norm on behavioral intention was not significant (SN → BI, β = −0.080, p = 0.192). Accordingly, H1, H2, H3, H4, H5, H7, H8, H9, and H10 were supported, whereas H6 was not supported. The negative sign of the SN → BI coefficient was therefore not interpreted substantively, because the effect was statistically negligible.

In terms of explanatory power, the model explained 58.4% of the variance in attitude (R² = 0.584), 50.7% in perceived value (R² = 0.507), 41.2% in perceived behavioral control (R² = 0.412), and 50.9% in behavioral intention (R² = 0.509), indicating that the structural model exhibited meaningful but incomplete explanatory capability for intention to use GAI fitness assistants.

### 4.5 Indirect effects testing

To formally examine the mediating mechanisms implied by RQ2 and the structural model, standardized specific indirect effects were tested using bootstrapped confidence intervals (see Table 6). Overall, all indirect paths reported in Table 6 were statistically significant, with 95% confidence intervals excluding zero. This pattern indicated that the non-significant direct path from subjective norm to behavioral intention did not imply the absence of social influence; rather, subjective norm was associated with intention through attitude, perceived behavioral control, and perceived value.

**Table 6:** Results of standardized specific indirect effects.

Indirect path	Standardized indirect effect	95% CI	p
SN → PBC → BI	0.044	[0.009, 0.093]	0.044
ISQ → ATT → BI	0.171	[0.072, 0.274]	0.001
SN → ATT → VAL → BI	0.071	[0.030, 0.118]	0.001
SN → ATT → BI	0.072	[0.020, 0.142]	0.020
ATT → VAL → BI	0.272	[0.181, 0.373]	<0.001
ISQ → ATT → VAL → BI	0.169	[0.105, 0.247]	<0.001
ISQ → ATT → VAL	0.379	[0.278, 0.489]	<0.001
SN → ATT → VAL	0.158	[0.071, 0.246]	<0.001
SN → VAL → BI	0.078	[0.026, 0.144]	0.010
ISQ → PBC → BI	0.079	[0.021, 0.139]	0.008

For subjective norm, the significant paths included SN → PBC → BI (standardized effect = 0.044, 95% CI [0.009, 0.093], p = 0.044), SN → ATT → BI (standardized effect = 0.072, 95% CI [0.020, 0.142], p = 0.020), SN → VAL → BI (standardized effect = 0.078, 95% CI [0.026, 0.144], p = 0.010), and the serial path SN → ATT → VAL → BI (standardized effect = 0.071, 95% CI [0.030, 0.118], p = 0.001). Thus, subjective norm operated through multiple mediated routes rather than as a direct normative-pressure pathway.

Indirect effects involving information system quality and attitude were also significant. ISQ was indirectly associated with behavioral intention through ATT (standardized effect = 0.171, 95% CI [0.072, 0.274], p = 0.001), through PBC (standardized effect = 0.079, 95% CI [0.021, 0.139], p = 0.008), and through the serial path ISQ → ATT → VAL → BI (standardized effect = 0.169, 95% CI [0.105, 0.247], p < 0.001). ATT also showed a significant indirect association with BI through VAL (standardized effect = 0.272, 95% CI [0.181, 0.373], p < 0.001), while the upstream indirect paths ISQ → ATT → VAL (standardized effect = 0.379, 95% CI [0.278, 0.489], p < 0.001) and SN → ATT → VAL (standardized effect = 0.158, 95% CI [0.071, 0.246], p < 0.001) further indicated that attitude contributed to the formation of perceived value.

### 4.6 Robustness check with control variables

**Fig 4:**
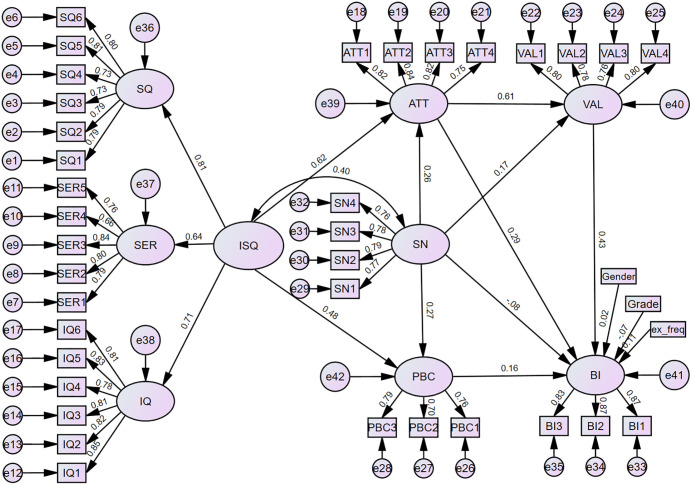
Structural model with control variables.

To account for differences in participants’ engagement levels and related experience, gender, education level, and exercise frequency were included as control variables. The purpose of incorporating these controls was to reduce potential confounding effects of demographic characteristics and exercise behavior differences on the relationships among the core constructs, thereby improving the accuracy and explanatory power of structural path estimates. The results (see Fig 4) showed that after controlling for gender, education level, and exercise frequency, the main path directions and significance levels among second-order information system quality (ISQ), subjective norm (SN), attitude (ATT), perceived behavioral control (PBC), and perceived value (VAL) remained consistent with those of the model without controls, and the overall fit indices did not exhibit any substantial changes. Further analyses indicated that the direct effects of gender, highest education level, and exercise frequency on behavioral intention (BI) were all non-significant. These findings suggested that the key structural relationships of the model did not change markedly across these control variables within the current sample.

## 5. Discussion

### 5.1 The role of information system quality

Second-order information system quality (ISQ) significantly promoted both attitude and perceived behavioral control (ISQ → ATT, β = 0.623; ISQ → PBC, β = 0.485), and system quality contributed the largest weight to the higher-order construct (0.805). This empirical result indicates that users first formed a broad judgment about whether GAI fitness assistants were stable, responsive, understandable, and supportive, and this judgment then shaped both favorable evaluations and perceived ability to use the assistant effectively.

This pattern is consistent with the IS Success Model and with prior digital health research showing that information, system, and service quality shape satisfaction, benefit perception, and intention [34,44,46]. It also aligns with research on conversational agents and large language models, where users are especially sensitive to fluency, response consistency, and the risk that plausible outputs may be inaccurate or unsupported [13,81,82].

In the GAI fitness context, quality is technology-specific because conversational interaction and iterative plan refinement are not merely interface conveniences; they shape exercise decisions with potential safety consequences. If an assistant hallucinates an exercise prescription, ignores injury history, misinterprets wearable data, or fails to explain intensity and recovery logic, users may perceive both lower value and higher injury risk. The practical implication is that developers should prioritize stable dialogue flows, wearable-data integration with clear consent, traceable explanations, risk warnings for unsafe exercise advice, and escalation pathways to human coaches or clinicians when the user’s condition exceeds the assistant’s safe boundary.

### 5.2 The role of subjective norm

Subjective norm had significant positive effects on attitude, perceived behavioral control, and perceived value (SN → ATT, β = 0.260; SN → PBC, β = 0.270; SN → VAL, β = 0.174), whereas its direct path to behavioral intention was not significant (SN → BI, p = 0.192). Empirically, social influence did not operate as a direct pressure to use the assistant; instead, it worked indirectly by shaping evaluations, perceived controllability, and value judgments. The formal indirect-effect tests further confirmed this interpretation, because the SN-related indirect paths through PBC, ATT, VAL, and the serial ATT → VAL route were all significant, whereas the direct SN → BI path remained non-significant.

This indirect pattern is broadly consistent with technology-adoption research showing that social influence often works through usefulness beliefs, attitude, and facilitating conditions rather than through a simple direct path [50,51]. Fitness-app studies have also shown that peer support, social features, and social comparison can influence exercise-related intentions and behavior by supporting self-regulation [26,28,83], while generative AI adoption studies in education similarly position subjective norm alongside attitude, perceived control, and AI literacy [38,52].

For GAI fitness assistants, the specific mechanism is informational rather than merely normative. Peer demonstrations, teacher or coach recommendations, and community discussions can provide prompt examples, safe-use cautions, and lessons about how to refine a plan after soreness, schedule changes, or wearable feedback. These forms of peer learning reduce trial-and-error costs and make perceived value more concrete. The implication is that promotion should emphasize support-oriented diffusion – such as prompt libraries, peer-shared cases, safety checklists, and coach-endorsed examples – rather than relying on social pressure or unsupported fear-of-missing-out narratives.

### 5.3 The role of attitude

Attitude exerted a significant direct effect on behavioral intention (ATT → BI, β = 0.275) and a stronger indirect pathway through perceived value (ATT → VAL, β = 0.608; VAL → BI, β = 0.447). This result shows that favorable evaluations of using GAI fitness assistants mattered in their own right, but they also became influential because they increased users’ belief that the assistant was worth the time, effort, cost, and risk involved. The bootstrapped indirect effect of ATT → VAL → BI was also significant, indicating that perceived value transmitted a substantial part of the association between favorable attitude and intention.

Prior TPB and generative AI adoption studies similarly identify attitude as a central antecedent of intention [37,38,52,53]. However, attitude differs from perceived value: attitude captures whether users evaluate the behavior positively, whereas perceived value captures whether benefits exceed sacrifices [39,40]. Modeling attitude as an antecedent of perceived value is therefore appropriate because positive affect, perceived convenience, and perceived usefulness can increase the salience of benefits while lowering the subjective burden of interaction effort.

In GAI fitness, attitude is fragile because a smooth conversational tone may mask hallucinated or unsafe exercise advice. Positive attitude is more likely to convert into value when the assistant explains why a plan is appropriate, admits uncertainty, uses wearable data transparently, and encourages human-coach escalation for pain, injury history, or medical-risk cases. The implication is that platforms should not rely only on enjoyable dialogue; they should design explainable and correctable interactions that let users revise prompts, compare alternatives, and understand the rationale behind load, frequency, progression, and recovery recommendations.

### 5.4 The role of perceived behavioral control

Perceived behavioral control had a significant but relatively modest effect on behavioral intention (PBC → BI, β = 0.163), and it was strengthened by both ISQ and SN. The empirical result suggests that perceived controllability functions as a feasibility threshold: users may value the assistant, but intention weakens when they doubt their ability to communicate goals, judge the safety of outputs, or integrate the assistant into daily training routines.

This result is consistent with TPB and AI adoption research emphasizing perceived capability, effort expectancy, self-efficacy, and facilitating conditions [15,16,47,48]. In generative AI adoption research, AI literacy and self-efficacy are also repeatedly identified as conditions that shape intention [52,55].

For GAI fitness assistants, PBC is technology-specific because users need prompt literacy, basic training knowledge, privacy and permission awareness, and the ability to interpret wearable-data-driven feedback. Users also need to know when a recommendation is too generic, too intense, or unsuitable for pain and injury conditions. The implication is that systems should provide onboarding, example prompts, adjustable plan templates, plain-language safety explanations, confidence or uncertainty cues, and simple routes to human coaching support, thereby turning perceived control into safer future-use intention rather than blind reliance.

### 5.5 The role of perceived value

Perceived value exhibited the strongest direct effect on behavioral intention (VAL → BI, β = 0.447). Empirically, users’ intention toward GAI fitness assistants was predicted most strongly by whether they believed the overall benefits of using the category outweighed the sacrifices involved.

This finding is consistent with value-based adoption research, which argues that intention depends on a trade-off between perceived benefits and perceived sacrifices [39,40]. It is also compatible with mHealth and fitness-app studies showing that usefulness, service experience, social support, and investment can influence adoption or future-use intention [18,26,28]. In generative AI chatbot services, perceived competence and warmth have likewise been linked to functional, social, and emotional value [54].

In the GAI fitness setting, perceived value combines distinctive benefits – conversational interaction, on-demand personalization, iterative plan refinement, motivational companionship, and possible integration with wearable data – with distinctive costs and risks, including privacy concerns, subscription costs, prompt effort, hallucinated or unsafe exercise advice, and injury risk. The implication is that providers should communicate value through verifiable outcomes and risk controls: transparent data use, explainable recommendations, clear safety boundaries, opt-in wearable integration, human-coach escalation, and pricing that matches the reliability and personalization actually delivered.

### 5.6 Model explanatory power and robustness

At the model level, the proposed framework explained 50.9% of the variance in behavioral intention, while the R² values for attitude (0.584), perceived value (0.507), and perceived behavioral control (0.412) were also meaningful. This empirical result indicates that integrating second-order information system quality, TPB mechanisms, and perceived value captured a substantial portion of intention formation, but it also leaves approximately 49.1% of behavioral intention unexplained.

Prior adoption studies similarly show that intention is rarely explained by a single theoretical lens; technological quality, social influence, trust, risk, effort, habit, and context-specific skills often work together [15,16,41]. For GAI fitness assistants, the unexplained variance is especially important because the technology category combines generative AI uncertainty with exercise safety, personal health data, and wearable-device ecosystems.

The omitted factors most likely include trust, perceived risk, privacy concerns, AI literacy, prompt self-efficacy, subscription cost, habit, wearable-device integration, prior fitness-app experience, and product-specific familiarity. The practical implication is that future models should treat the present framework as a meaningful baseline rather than a complete explanation. Longitudinal designs, usage logs, prompt and dialogue records, wearable indicators, and churn analyses would be needed to test whether these perceptions translate into actual use, safe training behavior, or discontinuance over time. Because the present data are cross-sectional and self-reported, the reported paths should be interpreted as theory-guided associations rather than causal effects.

## 6. Implications

### 6.1 Theoretical implications

The Information Systems Success Model and the Theory of Planned Behavior explain user adoption from the perspectives of technological quality evaluation and psychological decision-making mechanisms, respectively. However, in the digital health context of generative AI fitness assistants—characterized by high interactivity, substantial individual heterogeneity, and safety-related consequences—any single theory is often insufficient to capture the full chain from technology perceptions to intention formation. This study structurally integrated the IS Success Model with TPB and introduced perceived value as a key mediator, thereby developing an explanatory framework in which technological attributes, social cognition, and value trade-offs jointly explain intention to use. In doing so, the study extended the applicability boundaries and clarified the complementary relationship of these classic theories in generative-AI-enabled health behavior settings.

More specifically, this study consolidated information quality, system quality, and service quality into a second-order information system quality construct to capture users’ holistic judgments of the overall performance of GAI fitness assistants. It further demonstrated the significant effects of this higher-order quality construct on attitude and perceived behavioral control, highlighting the upstream role of quality perceptions in the intention-formation process. This specification not only responded to the integrated demands for stability, content reliability, and support assurance in fitness recommendation generation, but also provided a testable modeling approach for adopting higher-order quality constructs in future research on emerging generative AI applications. Accordingly, this study contributes to explaining category-level intention formation toward GAI fitness assistants in a safety-sensitive service context, without extending its claims to product-specific adoption, actual use, or post-adoption retention.

In addition, the findings showed that perceived value exerted the strongest explanatory power for intention to use, and that attitude and subjective norm indirectly promoted intention formation by enhancing value judgments. This pattern suggested that, in high-risk and sustained-engagement technology use, users’ decisions relied more on an overall weighing of benefits versus costs than on simple evaluations of liking or perceived usability. Meanwhile, subjective norm did not show a significant direct effect on intention to use but had significant effects on attitude, perceived behavioral control, and perceived value, indicating that social influence in this context was more likely to function as an antecedent source of cognitive cues and resource support rather than as a mechanism of direct normative pressure. These findings offered new empirical evidence regarding the boundary conditions of TPB’s social influence pathway in low-visibility, highly individualized health management behaviors. By formally testing the indirect effects, this study further clarified that subjective norm contributed to intention formation mainly through mediated routes, strengthening the theoretical argument that social influence in this context functions as informational and value-shaping support.

### 6.2 Practical implications

This study provides clear implications for the product design, operational strategies, and governance practices of generative AI fitness assistants. First, developers should treat improvements in second-order information system quality as foundational work for increasing adoption intention. In particular, system quality should be prioritized as the core source of overall quality by enhancing response speed, interaction stability, error tolerance, and process guidance to ensure a seamless conversational experience. This can help prevent training workflows from being disrupted by lag, interruptions, or complex operations, which would otherwise undermine users’ attitudes and perceived controllability. Second, information quality should be strengthened systematically by establishing content generation and verification mechanisms centered on recommendation accuracy, relevance, actionability, and contextual fit. For example, integrating movement-risk alerts, progressive overload rules, identification of individual constraints, and explanatory feedback can reduce uncertainty and safety concerns and improve users’ judgments of recommendation credibility and usefulness. Third, although service quality carries a relatively lower weight, it remains an important lever for reducing learning costs in the early stage of diffusion. Providing onboarding tutorials, prompt/example libraries, self-service troubleshooting for common issues, and, when necessary, human-AI collaborative support can reduce frustration and further enhance perceived behavioral control.

Second, operators should build the core growth logic around perceived value. Given that perceived value is the strongest driver of intention, platforms need to simultaneously strengthen the benefit side while lowering the cost side. On the benefit side, platforms should highlight gains from personalized plan generation, efficient dynamic adjustment, time and money savings, and experiential benefits derived from companion-like interaction. On the cost side, efforts should focus on reducing learning and communication burdens, privacy and data security concerns, and risk expectations associated with incorrect recommendations. Concretely, practices such as data minimization and transparent consent, traceable explanations for training recommendations, clear risk-boundary prompts, and appropriate referral mechanisms can increase certainty in the perceived return on investment, making it easier for users to form an overall judgment that the assistant is worth using in the future.

Third, managing subjective norms should shift from pressure-based promotion to support-oriented diffusion. Given the limited direct effect of subjective norm on intention, a more effective approach is to leverage communities and word of mouth to provide experiential cues and learning resources. Examples include building peer-shared training cases and dialogue templates, and encouraging users to present improvement processes and key precautions. This can translate social endorsement into more positive attitudes, stronger perceived controllability, and higher value judgments, rather than relying on others’ expectations alone. Finally, at the regulatory and industry levels, quality and value can be used as governance levers to promote safety and disclosure standards for generative-AI-based exercise recommendations, clarifying requirements for risk warnings, data protection, and responsibility boundaries. Such measures can reduce users’ perceived costs and uncertainty at the institutional level and provide external safeguards for the sustainable use of these tools in digital health contexts.

## 7. Limitations and future research

Although this study developed an integrative model based on second-order information system quality, the Theory of Planned Behavior, and perceived value, and provided a relatively systematic explanation of the mechanism underlying intention to use generative AI fitness assistants, several limitations remained and should be addressed in future research.

First, the sample was collected via an online survey. Although response-time screening, an attention-check item, and straight-lining detection were applied to enhance data quality, convenience sampling and self-selection bias were difficult to avoid. Individuals who were more familiar with generative AI or more engaged in digital fitness were more likely to participate, which may have inflated overall levels of positive attitudes and value judgments and limited the external validity of the findings for broader populations. In addition, the 342 valid responses for 35 measurement items yielded approximately 9.77 responses per item. This ratio is close to the 10:1 guideline but below more conservative 15:1 or 20:1 recommendations. Future studies could adopt stratified sampling or multi-channel recruitment to include participants with diverse ages, exercise experience, health conditions, and levels of digital skills, and could use bootstrap robustness checks or larger independent samples to strengthen confidence in the estimates.

Second, this study relied on cross-sectional self-reported data. Although the directional relationships among variables were theoretically grounded, strict causal inference remained limited. Moreover, behavioral intention was not equivalent to actual use, actual continued use, or churn, especially in the safety-sensitive context of fitness recommendation generation, where the intention-behavior link may be disrupted by unexpected injuries, fluctuations in time and resource availability, device constraints, privacy concerns, subscription costs, or risk-related incidents. Future research could incorporate longitudinal tracking, field experiments, or quasi-experimental designs, and combine real usage logs, dialogue records, training execution data, wearable-device indicators, and churn records to test the intention-to-behavior conversion pathway and to evaluate the actual effects of such tools on exercise adherence, training load management, and injury risk.

Third, the study had target-system ambiguity. The questionnaire did not collect product names, usage duration, frequency of use, subscription status, wearable-device ownership, or actual interaction records. As a result, the internal validity of product-specific interpretations is limited, and the findings should not be read as evidence about any particular system’s performance, retention, or user experience. Future studies could ask respondents to identify the target system, distinguish trial users from frequent users, and combine survey measures with platform logs or interview data to clarify how specific product features shape intention and behavior.

Fourth, this study modeled users’ core evaluations of generative AI fitness assistants using second-order information system quality, which helped simplify the structural model and capture holistic quality judgments, but may have obscured finer-grained differences among the first-order dimensions. For example, users may vary in sensitivity to system stability, information credibility, and support services, and the relative importance of these dimensions may shift across stages of use. Future studies could compare higher-order models with competing specifications, or adopt stage-based modeling and multi-group analyses to identify heterogeneity in dimension weights and path mechanisms across novice versus frequent users and across groups with different exercise goals, and to explore how quality dimensions influence perceived value and intention through distinct mediating processes.

Fifth, constrained by existing measurement systems, this study primarily relied on contextual adaptations of established items to measure several constructs. Although this approach ensured theoretical traceability, it may not have fully captured key psychological mechanisms in the high-risk and highly individualized setting of generative AI fitness assistants. For instance, trust, perceived risk, privacy and data security concerns, AI literacy, prompt self-efficacy, subscription cost, habit, prior fitness-app experience, judgments about model explainability and responsibility boundaries, risk expectations regarding the consequences of erroneous recommendations, wearable-device integration, and preferences for human-AI collaboration may all substantially shape value trade-offs and future-use intention. Future research could incorporate these variables into an extended framework, develop models that better reflect safety-sensitive decision logic, and create more context-specific measurement instruments to improve content validity and explanatory depth.

Finally, generative AI technologies evolve rapidly, and differences in product forms, model capabilities, data sources, and interaction modalities may shift the baseline of user experience, thereby altering the mechanisms through which quality perceptions and value judgments are formed. The conclusions of this study were most applicable to the current typical form of conversational, generative fitness recommendation services at the category level. Future research could conduct cross-platform, cross-modality, and cross-version comparative studies to examine the model’s applicability to service modes such as text-based generation, voice-based coaching, multimodal movement assessment, wearable-integrated coaching, and hybrid services integrating human coaches, and to identify relatively stable core drivers and transferable mechanisms under conditions of rapid technological change.

## 8. Conclusion

This study focused on generative AI fitness assistants as a digital health application category characterized by both high contextual adaptability and safety sensitivity. It conceptualized second-order information system quality from the IS Success Model as the technological foundation, applied the Theory of Planned Behavior (TPB) to explain the psychological pathways underlying intention formation, and further introduced perceived value to capture users’ overall trade-offs between benefits and costs. In doing so, the study developed an integrated framework for explaining category-level behavioral intention rather than product-specific post-adoption behavior. Guided by RQ1-RQ3, the proposed model was tested using survey data and covariance-based structural equation modeling (CB-SEM), yielding the following key conclusions.

For RQ1, the results indicated that second-order information system quality was significantly associated with more favorable attitude and greater perceived behavioral control. This finding suggested that, before forming intention to use, users first established a judgment basis regarding the system’s usability and controllability based on its overall performance. Further examination of the weight structure of the second-order construct showed that system quality contributed most strongly to overall quality perceptions, followed by information quality, while service quality also played a meaningful supporting role. This pattern implied that the stability and responsiveness of conversational interaction, the relevance and comprehensibility of generated recommendations, and the availability of problem-handling and support mechanisms jointly shaped users’ initial judgments of whether such a tool was reliable and worth investing in.

Regarding RQ2, subjective norm exerted significant positive effects on attitude, perceived behavioral control, and perceived value, whereas its direct effect on intention to use was not significant. This result suggested that social influence in this context was more likely to operate indirectly. Specifically, recommendations and experience sharing from peers, communities, or significant others functioned more as informational cues and learning resources: they reduced trial-and-error costs, strengthened users’ confidence in their own capabilities and resource conditions, and facilitated intention formation by improving value judgments, rather than directly driving future-use commitment through coercive normative pressure. Consistent with the formal indirect-effect tests, all specific SN-related indirect paths reported in Table 6 had 95% confidence intervals that excluded zero, supporting an indirect pattern rather than a direct normative-pressure mechanism.

For RQ3, the findings further revealed that among the factors influencing intention to use, perceived value served as the most critical direct predictor, while attitude and perceived behavioral control also played significant roles. This indicated that in the high-risk and highly individualized setting of fitness recommendation generation, users were unlikely to rely solely on liking or others’ recommendations when making decisions. Instead, they focused more on whether the assistant was worthwhile overall, namely, whether expected benefits could outweigh time and effort investments, privacy and safety concerns, and the potential consequences of recommendation errors. Overall, the model demonstrated meaningful but incomplete explanatory power, capturing 50.9% of the variance in intention while leaving substantial room for variables such as trust, perceived risk, privacy concerns, AI literacy, subscription cost, habit, wearable-device integration, and prior fitness-app experience.

Beyond the core paths, robustness checks further reinforced the credibility of the conclusions. After controlling for gender, highest education level, and exercise frequency, the directions and significance levels of the main relationships remained consistent, and the direct effects of the control variables on intention to use were not significant. These results indicated that the identified mechanisms were not driven by simple demographic differences or variations in exercise participation within the current sample, but instead reflected the structural roles of quality perceptions, attitudinal evaluations, perceived controllability, and value trade-offs. However, because the evidence is cross-sectional and self-reported, the conclusions should be read as associations among perceptions and intention rather than as causal effects or evidence of actual continued use.

Based on these findings, the study offered direct practical implications. For platforms and product designers, priority should be given to improving the stability and responsiveness of conversational interactions, enhancing the accuracy, contextual fit, and explainability of recommendation content, and building clear and accessible support and error-correction mechanisms to strengthen users’ quality perceptions. For university administrators and health education managers, promoting appropriate use of generative AI fitness assistants should go beyond tool introduction; it should also involve standardized usage guidance, risk communication, and data governance arrangements that help users reduce perceived costs and uncertainty, thereby increasing overall perceived value and fostering responsible future-use intention. Overall, as generative AI continues to reshape the boundaries of digital fitness and health management services, this study provided an integrative perspective for explaining intention formation and established a theoretical and empirical basis for future extensions across different platform forms, interaction modes, and user populations.

## Supporting information

S1 Appendix Questionnaire items. (DOCX)
