# Supplementary material for: Determinants of intention to use generative AI fitness assistants: Integrating the Theory of Planned Behavior, second-order information system quality, and perceived value
Source: PLoS One. 2026 Jul 9;21(7):e0353384. doi: 10.1371/journal.pone.0353384 (PMC13349156; doi:10.1371/journal.pone.0353384)
Supplement: S1 Appendix — (DOCX) [file pone.0353384.s001.docx]

**S1 Appendix. Questionnaire items.**

| **Construct** | **Items** | **Source** |
| --- | --- | --- |
| System Quality (SQ) | SQ1: The conversational function of the generative AI fitness assistant is reliable and stable. | Nelson et al., 2005 |
|  | SQ2: The generative AI fitness assistant responds to my requests quickly. |  |
|  | SQ3: I can easily access and use the generative AI fitness assistant to obtain training advice and information. |  |
|  | SQ4: When my training needs or conditions change, the generative AI fitness assistant can still flexibly adjust its recommendations. |  |
|  | SQ5: The generative AI fitness assistant can integrate scattered relevant information to provide more appropriate recommendations. |  |
|  | SQ6: Overall, the conversational function of the generative AI fitness assistant is of high quality. |  |
| Information Quality (IQ) | IQ1: Overall, the training advice provided by the generative AI fitness assistant is accurate. | Nelson et al., 2005 |
|  | IQ2: The training information I obtain from the generative AI fitness assistant rarely contains obvious errors. |  |
|  | IQ3: The information provided by the generative AI fitness assistant is sufficiently complete to support me in developing or adjusting a training plan. |  |
|  | IQ4: The training recommendations provided by the generative AI fitness assistant are updated in a timely manner and fit my current situation. |  |
|  | IQ5: The assistant’s responses are clearly expressed and well-structured, making them easy for me to understand and implement. |  |
|  | IQ6: Overall, the training information provided by the generative AI fitness assistant is of high quality. |  |
| Service Quality (SER) | SER1: When I encounter problems while using the generative AI fitness assistant, it provides clear guidance on how to handle them. | Parasuraman et al., 2005 |
|  | SER2: When I report an issue or request help, the platform/assistant responds promptly. |  |
|  | SER3: The help provided by the platform/assistant effectively resolves the problems I encounter. |  |
|  | SER4: The platform/assistant provides convenient support channels for me to obtain help. |  |
|  | SER5: The platform/assistant demonstrates professionalism and responsibility when providing support, which increases my confidence in the outcomes. |  |
| Perceived Behavioral Control (PBC) | PBC1: It is easy for me to learn how to use the generative AI fitness assistant. | Fishbein & Ajzen, 2011; Van Lange et al., 2012 |
|  | PBC2: In my daily workout arrangements, it is easy for me to have the generative AI fitness assistant help me develop a training plan. |  |
|  | PBC3: When I need to adjust my training plan, it is easy for me to use the generative AI fitness assistant to obtain recommendations. |  |
| Attitude (ATT) | ATT1: I think using the generative AI fitness assistant to develop a training plan is a wise choice. | Compeau & Higgins, 1995 |
|  | ATT2: I find it enjoyable to use the generative AI fitness assistant to support my workouts. |  |
|  | ATT3: I find it interesting to use the generative AI fitness assistant to help me analyze training-related issues. |  |
|  | ATT4: I really enjoy the convenience brought by using the generative AI fitness assistant. |  |
| Perceived Value (VAL) | VAL1: Compared with the costs I incur to use the generative AI fitness assistant, the benefits I gain are worthwhile. | Kim et al., 2007 |
|  | VAL2: Compared with the effort I need to invest, the assistance provided by the generative AI fitness assistant is beneficial to me. |  |
|  | VAL3: Compared with the time I need to spend, using the generative AI fitness assistant is worth it for me. |  |
|  | VAL4: Overall, using the generative AI fitness assistant provides me with high value. |  |
| Subjective Norm (SN) | SN1: My parents support me in using the generative AI fitness assistant to develop a training plan. | Ajzen, 1985 |
|  | SN2: People who are important to me think that I should use the generative AI fitness assistant to develop a training plan. |  |
|  | SN3: My classmates think that, in order to exercise better, it is necessary for me to use the generative AI fitness assistant to develop a training plan. |  |
|  | SN4: My physical education teacher thinks that it is necessary for me to use the generative AI fitness assistant to assist in developing a training plan. |  |
| Behavioral Intention (BI) | BI1: I intend to use the generative AI fitness assistant to develop a training plan. | Taylor & Todd, 1995 |
|  | BI2: I intend to use the generative AI fitness assistant frequently. |  |
|  | BI3: If it is available in the future, I am willing to continue using the generative AI fitness assistant to support my fitness training. |  |

Note: After the pilot test, IQ3 and SER4 were deleted, and the remaining items were renumbered; this table presents the final version.
